# Associations of Medications With Lower Odds of Typical COVID-19 Symptoms: Cross-Sectional Symptom Surveillance Study

**DOI:** 10.2196/22521

**Published:** 2020-12-14

**Authors:** Dietmar Urbach, Friedemann Awiszus, Sven Leiß, Tamsin Venton, Alexander Vincent De Specht, Christian Apfelbacher

**Affiliations:** 1 Helios-Klinikum Gifhorn Gifhorn Germany; 2 Department of Orthopaedic Surgery Otto von Guericke University Magdeburg Germany; 3 Gutingi-Medical Outpatient Clinic Göttingen Germany; 4 Teign Estuary Medical Group Teignmouth United Kingdom; 5 University of Potsdam Potsdam Germany; 6 Institute of Social Medicine and Health Systems Research Otto von Guericke University Magdeburg Germany

**Keywords:** COVID-19, SARS-CoV-2, statins, antihypertensives, surveillance, hydroxymethyl-glutaryl-coenzyme A reductase inhibitors, online survey

## Abstract

**Background:**

As the COVID-19 pandemic continues to spread across the globe, the search for an effective medication to treat the symptoms of COVID-19 continues as well. It would be desirable to identify a medication that is already in use for another condition and whose side effect profile and safety data are already known and approved.

**Objective:**

The objective of this study was to evaluate the effect of different medications on typical COVID-19 symptoms by using data from an online surveillance survey.

**Methods:**

Between early April and late-July 2020, a total of 3654 individuals in Lower Saxony, Germany, participated in an online symptom-tracking survey conducted through the app covid-nein-danke.de. The questionnaire comprised items on typical COVID-19 symptoms, age range, gender, employment in patient-facing healthcare, housing status, postal code, previous illnesses, permanent medication, vaccination status, results of reverse transcription polymerase chain reaction (RT-PCR) and antibody tests for COVID-19 diagnosis, and consequent COVID-19 treatment if applicable. Odds ratio estimates with corresponding 95% CIs were computed for each medication and symptom by using logistic regression models.

**Results:**

Data analysis suggested a statistically significant inverse relationship between typical COVID-19 symptoms self-reported by the participants and self-reported statin therapy and, to a lesser extent, antihypertensive therapy. When COVID-19 diagnosis was based on restrictive symptom criteria (ie, presence of 4 out of 7 symptoms) or a positive RT-PCR test, a statistically significant association was found solely for statins (odds ratio 0.28, 95% CI 0.1-0.78).

**Conclusions:**

Individuals taking statin medication are more likely to have asymptomatic COVID-19, in which case they may be at an increased risk of transmitting the disease unknowingly. We suggest that the results of this study be incorporated into symptoms-based surveillance and decision-making protocols in regard to COVID-19 management. Whether statin therapy has a beneficial effect in combating COVID-19 cannot be deduced based on our findings and should be investigated by further study.

**Trial Registration:**

German Clinical Trials Register DRKS00022185; https://www.drks.de/drks_web/navigate.do?navigationId=trial.HTML&TRIAL_ID=DRKS00022185; World Health Organization International Clinical Trials Registry Platform U1111-1252-6946

## Introduction

More than 11 months have passed since SARS-CoV-2 infection was first detected in Wuhan, China, but the worldwide incidence of COVID-19 is still increasing [1]. Despite widespread research efforts, no curative therapies for COVID-19 have been established, and a safe vaccine is unlikely to become available before 2021. In Germany, the first known case of infection with SARS-CoV-2 was reported in the end of January 2020, and a peak of new infections was noted during March and April [2,3].

Early epidemiological studies of the disease demonstrated that age and preexisting medical conditions, in particular cardiopulmonary diseases, are associated with a high mortality rate in patients hospitalized due to COVID-19 [4]. However, it remains unclear why some individuals within high-risk groups have severe disease, whereas others do not. Moreover, the role of medication, as well as vaccination status, in preventing patients with COVID-19 from becoming severely ill remains debated [5,6]. With regard to the use of statins (also known as hydroxymethylglutaryl-coenzyme A reductase inhibitors) in particular, 3 recent studies found these medications have a direct effect on COVID-19 severity and outcomes [7-9].

In addition, there was insufficient surveillance of the disease when the infection spread to Germany. COVID-19 surveillance testing based on reverse transcription polymerase chain reaction (RT-PCR) results was initiated by Robert Koch Institution (RKI) in Germany. However, due to limited testing resources, only a small cohort of people were included during the first wave of COVID-19. Furthermore, no surveillance system based on typical COVID-19 symptoms had been established in Germany at that time.

Many management decisions and recommendations regarding COVID-19 are based on the presence of typical symptoms. These include screening for patients with suspected COVID-19, an indication algorithm for PCR testing, and recommendations for self-isolation. In addition, symptom tracking at the regional level has been used to identify increases in infection rate [10,11]. At the beginning of this study, the following symptoms were commonly observed in patients with laboratory-confirmed COVID-19: fever, dyspnea, dry cough, sore throat, myalgia, headache, ageusia, and anosmia [4,12].

Therefore, to address the abovementioned research challenges and test a symptom-based regional surveillance system, we decided to combine an online symptom-tracking app with the elicitation of medical data that might have an impact on COVID-19 cases. The survey was rolled out at the end of March 2020 as a pilot project in Lower Saxony, Germany. At the same time, a symptom-tracking app was distributed in the UK [10]. Later, symptom trackers were established successfully in more regions [13].

Thus, the aim of this study was to evaluate the effects of different medications on typical COVID-19 symptoms by using data from our surveillance survey.

## Methods

### Study Recruitment

For the pilot study, we recruited participants from the administrative district of Gifhorn in the German federal state of Lower Saxony, by advertising via regional newspaper articles, a YouTube video, social media, and regional broadcasts.

### Data Collection

Data were collected using an online questionnaire implemented by the browser-based app “covid-nein-danke.de” [14], which was developed between March 10 and 27, 2020, by 2 authors (SL and DU). For this evaluation, we considered data entered by participants from April 2 to July 20, 2020.

The questionnaires were completed using a computer or an internet-enabled mobile phone or tablet device. Owing to data protection requirements, the app was developed as a browser-based app that did not require users to download a program. Technically, the study was based on a single-page app written in JavaScript and a highly scalable cloud. To address the estimated high data volumes and make the questionnaire adaptable for changes, a NoSQL (non–structured query language) database was used.

The survey was described and initiated under the URL covid-nein-danke.de [14]. The overall time taken to complete the survey was approximately 3-5 minutes. After finishing the survey, a 7-digit code was randomly generated and provided to the user for follow-up surveys. The app worked without user “tracking”—neither their email address nor IP address was stored. Users were not identified in any way; thus, data collection was completely anonymous.

### Questionnaire Items

The questionnaire contained items on age range, gender (male, female, and nonbinary for those reporting their gender as diverse), employment in patient-facing health care, housing status, postal code, previous illnesses, permanent medication, vaccination status, and symptoms. It also included the results of any COVID-19 PCR or antibody tests and any COVID-19 treatment received. More specifically, the following new COVID-19 symptoms were assessed: dry cough, increased body temperature or fever (>37.5°C), shortness of breath, muscle or joint pain, sore throat, headache, and loss of smell or taste. The participants confirmed “yes” or “no” for each question.

For the question “Do you take regular medication?” if the participant responded “yes,” a list of medications was displayed for the participant to select by using a “yes-no” slider. Medications listed included cholesterol-lowering medication (eg, simvastatin), nonsteroid anti-inflammatory drugs (NSAIDs, such as ibuprofen and diclofenac), thyroid medication (eg, levothyroxine), omeprazole/pantoprazole, metamizole, antihypertensives, furosemide or hydrochlorothiazide (HCT), cortisone, disease-modifying antirheumatic drugs (DMARDs, eg, methotrexate, biologics, hydroxychloroquine, and antihistamines). If “antihypertensives” were chosen, participants were asked to choose from the following selection: ramipril, beta-blockers metoprolol, bisoprolol, and amlodipine. Participants could also provide further information on medications not explicitly requested in a free-text field.

### Statistical Analysis

The raw data were transformed from a JavaScript Object Notation format to a rational data format for further evaluation. Data were analyzed using SAS/STAT software (SAS Institute Inc.). To investigate associations between typical COVID-19 symptoms and medical characteristics, we ran logistic regression models (logit model). Odds ratio (OR) estimates with corresponding 95% CIs were computed for each medication and symptom. Based on the assumption that the presence of 4 out of 7 typical symptoms indicates COVID-19, the association of COVID-19 with concomitant medication intake was similarly determined.

### Ethics and Informed Consent

The online study was approved by the ethics committee of the Otto-von-Guericke-University Magdeburg (Ref. 65-20). This study is registered with the German Clinical Trial Register (No. DRKS00022185) and World Health Organization (WHO) International Clinical Trials Registry Platform U1111-1252-6946.

An overview of the aims of this survey is provided on the homepage of the app’s website [14]. Furthermore, a link directs participants to the details of the survey and data evaluation. Before the questionnaire could be opened, participants were required to agree to the data privacy policy according to European General Data Protection Regulation. Another page informed the participants about data storage, processing, and evaluation. They were also informed about their rights and that participation was not associated with any direct benefit or compensation. Furthermore, the participants were required to confirm that they were over 18 years old before proceeding to the questionnaire.

## Results

### Epidemiological Data

From April 2 to July 20, a total of 3990 people participated in the online survey. From these, data from 3654 participants were further evaluated. Data from the remaining 336 participants were excluded owing to incomplete symptom data entry. The peak age ranges were 50-59 years for female participants and 60-69 years for male participants. Overall, there were more female participants (2250/3654, 61.6%) than male participants (1394/3654, 38.1%; Table 1). 

**Table 1:** Gender-specific age-range distribution for participants of the online survey conducted between April and July 2020.

Age range, years	Gender, n (%)			
	Male	Female	Nonbinary	Not stated
18-29	97 (7.0)	235 (10.4)	1 (33.3)	N/A^a^
30-39	154 (11.0)	359 (16.0)	N/A	1 (14.3)
40-49	234 (16.8)	471 (20.9)	N/A	N/A
50-59	339 (24.3)	619 (27.5)	1 (33.3)	3 (42.9)
60-69	342 (24.5)	422 (18.8)	1 (33.3)	2 (28.6)
70-79	186 (13.3)	117 (5.2)	0 (0.0)	1 (14.3)
80-89	42 (3.0)	25 (1.1)	0 (0.0)	0 (0.0)
>90	0 (0.0)	2 (0.1)	N/A	N/A

^a^N/A: not applicable.

Of the 3654 respondents, 99 were tested by RT-PCR at the same time as they participated in the survey. Of these, 16.16% (16/99) participants tested positive for SARS-CoV-2, corresponding to only 0.44% (16/3654) of all survey participants.

### Medications

We found significantly lower odds of self-reported symptoms for COVID-19 among participants taking statin, antihypertensive, and diuretic medications. In contrast, significantly higher odds of self-reported COVID-19 symptoms was associated with participants taking DMARDs, metamizole, and cortisone. Results of the logistic regression analysis for specific medications are presented in Table 2. The findings show a significant association between certain medications and either higher or lower self-reported odds of COVID-19 symptoms.

**Table 2:** Strength of association between medication and odds of typical COVID-19 symptoms that were self-reported (N=3645).

Medication	Number of participants taking medication, n (%)	COVID-19 symptom, odds ratio estimate (CI^a^)						
		Headache	Fever	Loss of smell or taste	Sore throat	Shortness of breath	Joint-muscle pain	Dry cough
Statins	358 (10)	*0.42* * (0.28-0.64)* ^b^	0.43 (0.13-1.45)	0.67 (0.29-1.53)	*0.54* * (0.35-0.85)*	1.15 (0.71-1.87)	0.87 (0.56-1.34)	*0.61* * (0.43-0.88)*
NSAIDs^c^	212 (6)	*1.93* * (1.38-2.7)*	0.4 (0.09-1.67)	0.91 (0.36-2.34)	*1.58* * (1.08-2.31)*	*2.36* * (1.48-3.75)*	*2.1* * (1.41-3.13)*	*1.57* * (1.10-2.23)*
Thyroid medication	585 (16)	1.13 (0.89-1.43)	0.63 (0.30-1.32)	0.62 (0.33-1.19)	0.78 (0.59-1.04)	0.84 (0.56-1.26)	1.02 (0.75-1.39)	0.93 (0.72-1.20)
Omeprazole/ pantoprazole	399 (11)	0.95 (0.70-1.3)	1.62 (0.77-3.42)	0.71 (0.33-1.52)	1.15 (0.83-1.61)	0.93 (0.58-1.47)	1.07 (0.74-1.56)	0.81 (0.59-1.12)
Metamizole	127 (3)	*2.31* * (1.50-3.55)*	1.47 (0.47-4.56)	*2.91* * (1.17-7.22)*	1.44 (0.87-2.40)	*2.62* * (1.45-4.73)*	*2.44* * (1.47-4.05)*	*1.74* * (1.10-2.74)*
Antihypertensives (all)	1094 (30)	0.76 (0.55-1.04)	0.48 (0.18-1.26)	1.18 (0.59-2.36)	*0.65* * (0.45-0.93)*	1.44 (0.92-2.26)	0.96 (0.65-1.42)	1.22 (0.90-1.65)
Furosemide or hydrochlorothiazide	192 (5)	0.71 (0.44-1.15)	0.58 (0.13-2.64)	0.92 (0.35-2.41)	*0.39* * (0.20-0.75)*	0.59 (0.29-1.21)	0.71 (0.40-1.27)	1.02 (0.67-1.57)
Cortisone	169 (5)	1.24 (0.83-1.86)	1.47 (0.54-3.98)	0.96 (0.33-2.79)	1.26 (0.81-1.97)	*1.83* * (1.05-3.18)*	*1.8* * (1.12-2.90)*	1.26 (0.82-1.92)
DMARDs^d^	56 (2)	0.5 (0.23-1.09)	0.66 (0.08-5.30)	0.82 (0.11-6.33)	1.66 (0.85-3.23)	1.6 (0.69-3.76)	0.54 (0.20-1.45)	1.01 (0.49-2.05)
Antihistamines	239 (7)	1.31 (0.94-1.82)	0.73 (0.26-2.07)	0.98 (0.42-2.32)	1.12 (0.77-1.65)	1.22 (0.72-2.05)	0.91 (0.57-1.45)	0.97 (0.67-1.41)
Biologics	142 (4)	*1.75* * (1.18-2.59)*	1.32 (0.46-3.79)	0.24 (0.03-1.79)	0.85 (0.51-1.43)	0.57 (0.25-1.28)	0.92 (0.52-1.63)	0.95 (0.59-1.52)
Hydroxy- chloroquine	28 (1)	1.8 (0.76-4.25)	1.72 (0.22-13.8)	—^e^	1.66 (0.66-4.16)	0.97 (0.26-3.54)	0.49 (0.11-2.15)	1.44 (0.59-3.54)
Subgroup ARBs^f^	39 (1)	0.87 (0.33-2.34)	—	—	0.52 (0.12-2.21)	1.21 (0.35-4.14)	0.9 (0.26-3.05)	1.14 (0.48-2.67)
Subgroup ACEIs^g^ (eg, ramipril)	412 (11)	0.94 (0.65-1.37)	0.93 (0.31-2.83)	*0.38* * (0.15-0.93)*	1.16 (0.76-1.75)	0.85 (0.50-1.44)	1.12 (0.72-1.74)	0.93 (0.66-1.31)
Subgroup beta-blockers	437 (12)	0.95 (0.65-1.37)	1.9 (0.66-5.46)	2.1 (1.01-4.35)	1.24 (0.82-1.87)	0.82 (0.49-1.39)	1.05 (0.68-1.64)	1.14 (0.81-1.59)
Subgroup calcium-channel blockers (eg, amlodipine)	209 (6)	1.35 (0.88-2.06)	1.14 (0.31-4.16)	1.49 (0.65-3.39)	1.23 (0.75-2.00)	0.74 (0.38-1.46)	0.66 (0.36-1.19)	1.06 (0.70-1.59)

^a^Lower and upper limits of 95% Wald confidence interval.

^b^Italic text indicates statistically significant values.

^c^NSAIDs: nonsteroidal anti-inflammatory drugs.

^d^DMARDs: disease-modifying antirheumatic drugs.

^e^Not applicable (ie, numbers were too low to be calculated).

^f^ARBs: angiotensin receptor blocker.

^g^ACEIs: angiotensin converting enzyme inhibitors.

#### Statins

Of the 3654 participants, 358 (9.8%) indicated that they were taking regular statin medications. The regular intake of statin medication was significantly associated with lower odds of those participants reporting symptoms such as sore throat, dry cough, and headache. The odds of loss of smell or taste, as well as fever, were also decreased among these participants; however, it did not reach statistical significance. No association was found between statin medication and the odds of shortness of breath and joint or muscle pain. Statistically significant associations were found for both male and female participants with respect to headache, and only for female participants with respect to sore throat despite a lowered statistical power through gender stratification (Table 3).

**Table 3:** Gender-stratified strength of association between statin administration and typical COVID-19 symptoms (n=358). Nonbinary participants (n=3) and those not reporting their gender (n=7) were not included in the analysis due to low numbers.

Gender (n)	COVID-19 symptom, odds ratio estimate (95% CI^a^)						
	Headache	Fever	Loss of smell or taste	Sore throat	Shortness of breath	Joint or muscle pain	Dry cough
Female (2250)	*0.3* ^b^ ** * (0.15-0.59)*	0.63 (0.15-2.61)	0.85 (0.26-2.76)	*0.27* * (0.12-0.61)*	1.28 (0.63-2.57)	0.77 (0.38-1.53)	0.84 (0.5-1.42)
Male (1394)	*0.58* * (0.35-0.95)*	0.21 (0.03-1.58)	0.68 (0.24-1.95)	0.73 (0.44-1.2)	1.23 (0.67-2.25)	0.97 (0.59-1.6)	0.65 (0.41-1.02)

^a^Lower and upper limits of 95% Wald confidence interval.

^b^Italic text indicates statistically significant values.

#### Antihypertensives

Of the 3654 participants, 1094 (29.9%) indicated that they were taking regular antihypertensive medication. These participants had significantly lower odds of reporting sore throat. The odds of reporting symptoms such as fever and headache were also decreased in this group, but these associations did not reach statistical significance. Participants taking angiotensin converting enzyme inhibitors (ACEIs) were found to have significantly lower odds of reporting loss of smell or taste.

#### Furosemide or HCT

In all, 192 of 3654 (5.3%) participants were taking diuretics (ie, either furosemide or HCT). Diuretic intake was significantly associated with lower odds of these participants reporting sore throat.

#### NSAID and Metamizole

NSAID medication was taken by 212 (5.8%) participants and metamizole, by 127 (3.5%) of all 3654 participants. Participants using NSAIDs had significantly higher odds of reporting typical COVID-19 symptoms such as headache, sore throat, shortness of breath, joint or muscle pain, and dry cough. In contrast, metamizole use was associated with lower odds of reported headache, loss of smell or taste, shortness of breath, joint or muscle pain, and dry cough. The numbers and corresponding percentages of symptoms observed for each medication used for COVID-19 symptom treatment are shown in Table S1 in Multimedia Appendix 1.

By making a presumptive diagnosis of COVID-19, either by a positive RT-PCR test or based on the presence of 4 out of 7 positive symptoms, we were able to detect 142 cases of infections among the 3654 participants. Furthermore, statistical analyses revealed a marked inverse association between COVID-19 cases and statin use with an OR of 0.28 (95% CI 0.1-0.78). All other medications did not show a statistically significant association (Table 4).

**Table 4:** Strength of association between statins and presumed COVID-19 diagnosis based on a positive reverse transcription polymerase chain reaction test (n=16) or presence of symptoms (minimum 4 out of 7 typical symptoms, n=126).

Medication	Odds ratio estimates (95% CI^a^)
Statins	*0.28* ^b^ *(0.10-0.78)*
NSAIDs^c^ (eg, diclofenac. ibuprofen)	1.75 (0.93-3.27)
Thyroid medication	1.01 (0.64-1.62)
Omeprazole/ pantoprazole	1.10 (0.61-2.00)
Metamizole	1.75 (0.77-3.97)
Antihypertensives (all)	0.63 (0.32-1.24)
Furosemide or hydrochlorothiazide	0.24 (0.06-1.03)
Cortisone	1.83 (0.91-3.66)
DMARDs^d^	0.90 (0.25-3.23)
Antihistamines	1.10 (0.58-2.08)
Biologics	0.30 (0.07-1.24)
Hydrochloroquine	1.33 (0.28-6.24)
Subgroup ARBs^e^ sartane	1.19 (0.15-9.25)
Subgroup ACEIs^f^ (eg, ramipril)	1.17 (0.55-2.47)
Subgroup beta-blockers	0.90 (0.25-3.23)
Subgroup calcium-channel blockers (eg, amlodipine)	1.10 (0.58-2.08)

^a^Lower and upper 95% Wald confidence intervals.

^b^Italic text indicates statistically significant values.

^c^NSAIDs: nonsteroidal anti-inflammatory drugs.

^d^DMARDs: disease-modifying antirheumatic drugs.

^e^ARBs: angiotensin receptor blocker.

^f^ACEIs: angiotensin converting enzyme inhibitors.

## Discussion

### Principal Findings

The main finding of our study is a statistically significant inverse relationship between self-reported symptoms typical for COVID-19 and statin therapy and, to a lesser extent, antihypertensive therapy. As the world continues to search for a medication to cure or attenuate COVID-19, it would be desirable, and fortuitous, to identify such a medication that is already in use for another condition and whose side effect profile and safety data are already known and approved. In the search for such medication, we combined a COVID-19 symptom surveillance survey with a prospective observational study in which participants’ medication intake was assessed.

COVID-19 was assumed based on a positive RT-PCR test, hospital admission, professional treatment, or presence of a minimum of 4 out of 7 typical symptoms. Fortunately, the incidence of confirmed COVID-19 cases was low in the study area (Lower Saxony, Germany). During the study period, RKI recorded 208 cases based on positive RT-PCR tests and 4 deaths due to COVID-19 in this region [2]. Nevertheless, the statistical evaluation of the data demonstrated significant results regarding typical COVID-19 symptoms and medication intake.

Our prospective surveillance survey asked participants to indicate any presence of symptoms, dry cough, sore throat, fever, joint or muscle pain, shortness of breath, loss of smell or taste, and headache, only if they were new occurrences. On the initiation of the survey at the beginning of March, gastrointestinal symptoms were considered an uncommon symptom for COVID-19 [4]. Therefore, we did not include gastrointestinal symptoms such as abdominal cramping or diarrhea in our analysis. These symptoms, however, have been incorporated into the questionnaire of the ongoing survey. Of all the participant characteristics collected, we statistically evaluated the role of gender in detail because age and gender play an important role in the mortality and severity of COVID-19 [15]. Further stratification based on other characteristics would have led to small subcohorts hampering meaningful statistical analyses.

A Cochrane review [12] found 6 symptoms in at least one study with a sensitivity of more than 50%; these included cough, sore throat, fever, myalgia or arthralgia, fatigue, and headache. Of these symptoms, fever, joint or muscle pain, fatigue, and headache were considered red flags as their specificity was greater than 90%, resulting in a positive likelihood ratio of at least 5 for COVID-19 [12]. It was hypothesized that a combination of symptoms could lead to an increase in sensitivity and specificity. However, they found no study that had assessed combinations of different symptoms. As we were unable to use an evidence base to weigh the sensitivity and specificity of individual symptoms, we selected 4 out of the 7 symptoms to define symptomatic COVID-19.

We found that taking medication such as NSAIDs as well as metamizole was significantly associated with higher odds for most COVID-19 symptoms evaluated. This is an unsurprising finding given that NSAIDs and metamizole are typical medications for treating such symptoms. Moreover, cortisone was associated with a significantly higher self-reported odds of dry cough. It can be postulated that patients with chronic obstructive pulmonary disease take cortisone more frequently and present with this symptom more often. Intake of biologics was found to be significantly associated with higher odds of headache. Headache is a known side effect of these medications, which may explain these statistical results. Nevertheless, these findings are important as positive indicators to the reliability of the data reported by the participants.

Of the medications associated with lower odds of self-reported typical COVID-19 symptoms, statins showed the most distinct results. Most common symptoms reported by participants using statins were dry cough, sore throat, and headache. In addition, statins were associated with lower odds of fever, joint or muscle pain, and loss of smell or taste. However, the latter associations did not reach statistical significance.

When we defined a COVID-19 diagnosis based on the presence of 4 of 7 symptoms or a positive RT-PCR test, the result was more obvious with an OR of 0.28 (95% CI 0.1-0.78). However, we did not find such significant associations between COVID-19 symptoms and other evaluated medications.

Based on our findings, we suggest 2 most likely explanations for the observed association between statin use and the lower odds of symptoms suggestive of COVID-19. First, statin therapy may either prevent SARS-CoV-2 infection or lower the symptom burden of COVID-19. This hypothesis is in line with 3 recent clinical studies. In a retrospective study of 154 nursing home residents with COVID-19, statin therapy was significantly associated with the absence of symptoms [8]. The researchers defined COVID-19 by either a positive PCR test or the presence of typical COVID-19 symptoms. Two other studies support the direct influence of statin use on COVID-19; they found a significantly lower disease severity among hospitalized patients with COVID-19 [9] and lower mortality among those who received concomitant statin medication [7]. At first sight, it seems implausible that taking statin medication could prevent a SARS-CoV-2 infection. However, a direct protective effect of statins against SARS-CoV-2 infection was recently proposed by Reiner et al [16], using a molecular docking study. From our results so far, it would be premature to assess whether statins have a preventative effect. We hope that our prospective study will shed further light on this potential use of statins when more participants undergo PCR testing.

The second possible explanation for our finding is that the encountered symptoms are independent of COVID-19. The evaluated symptoms are recognized as typical for COVID-19, but they also overlap with other illnesses such as allergies, non–COVID-19 flu-like viral infections, or migraine. Statins might act on those symptoms independently of COVID-19. In this context, it was shown that statins in combination with vitamin D can reduce migraine [17]. Furthermore, the results of previous studies point to the fact that statins are an option for treating the symptoms of influenza [18,19] and pneumonia [20-22]. In summary, it is already accepted that statins have pleiotropic, anti-inflammatory, and immunomodulatory effects [23], which would explain our findings. It is also possible that a combination of both explanations, that is, an anti-inflammatory effect on both COVID-19 and non–COVID-19 illness, could explain the finding of significant associations between statins and lower self-reported odds of typical COVID-19 symptoms.

Our study findings also suggest that antihypertensives were associated with a significantly lower self-reported odds of dry cough. A similar trend in decreased self-reporting of fever and headache was also observed. Statistical analyses of the subgroups ARBs, ACEIs, beta-blockers, and calcium-channel antagonists led to insignificant results except for an association between ACEIs and loss of smell or taste (OR 0.38, 95% CI 0.15-0.93). This finding might be of interest, as loss of smell and taste have recently been proposed as cardinal symptoms for COVID-19 [13]. As the subgroups were very small, the latter results must be interpreted with caution. The intake of antidiuretics, furosemide and HCT, was associated with lower odds of sore throat but no reduction in the other symptoms. We have no explanation for this association, neither from our data nor from the literature.

Regardless of whether statins and antihypertensives act on symptoms linked to COVID-19, or whether they act on symptoms independent of COVID-19, we must keep in mind that those symptoms might be masked by this medication. Surveillance recommendations and medical assessments based on typical symptoms may not be reliable in patients taking statins and antihypertensives. Furthermore, individuals receiving statin therapy may be more likely to have asymptomatic infection and therefore at a greater risk of transmitting the infection unknowingly.

Our results should not be interpreted as a recommendation to take statins or hypertensive drugs for prevention of COVID-19 or to reduce disease severity. We are mindful of the fact that large studies have shown no positive effect of statins in intensive care patients and in sepsis-associated acute respiratory distress syndrome [24,25]. Furthermore, a large study has been discontinued because of the lack of benefit and evidence of significant early renal and liver failure while taking rosuvastatin medication [26]. Nevertheless, our data support the hypothesis that statins and antihypertensives may play a role in COVID-19 treatment and emphasize the potential value of ongoing clinical studies (eg, NCT04348695, NCT04343001, and NCT04351581).

### Study Limitations

Because of the nature of the survey, there are a few limitations to the study. First, the results are based on a self-selected group who are not necessarily representative of the general population. Second, self-assessment of symptoms is purely subjective. Nevertheless, we found plausible data from the NSAID, metamizole, and cortisone medication groups, which indicated that the participants completed symptom reporting seriously. Although very unlikely, we cannot rule out, that participants repeated the survey more than once because of its anonymous nature.

### Conclusions

The exact association between statin medications and the outcome of reduced symptoms in the study population is uncertain. It is possible that statins have a therapeutic or preventive effect on COVID-19; it is equally possible that uninfected individuals receiving these drugs have a lower prevalence of symptoms unrelated to COVID-19. Thus, future studies are needed to evaluate the potential benefits of statins in patients with COVID-19.

Furthermore, we propose that statin therapy masks or reduces the symptoms in patients with SARS-CoV-2 infection. As such, patients receiving statin therapy may be more likely to have asymptomatic COVID-19, in which case they are at an increased risk of transmitting it unknowingly. We suggest that our study results are incorporated in the symptoms-based surveillance and decision-making protocols for COVID-19 management.

## Appendix

Multimedia Appendix 1 Symptoms observed for different medications used for COVID-19 treatment.

## Abbreviations

ARB: angiotensin receptor blocker
ACEI: angiotensin converting enzyme inhibitor
HCT: hydrochlorothiazide
NSAID: nonsteroidal anti-inflammatory drug
DMARD: disease-modifying antirheumatic drug
RKI: Robert Koch Institute, Germany
RT-PCR: reverse transcription polymerase chain reaction
WHO: World Health Organization
