# Supplementary material for: Associations of Medications With Lower Odds of Typical COVID-19 Symptoms: Cross-Sectional Symptom Surveillance Study
Source: JMIR Public Health Surveill. 2020 Dec 14;6(4):e22521. doi: 10.2196/22521 (PMC7744147; doi:10.2196/22521)
Supplement: Multimedia Appendix 1 [file publichealth_v6i4e22521_app1.docx]

# Table S1

The table shows the percentages of symptoms for each medication. The total number of participants was 3654.

| **Symptoms**  **Medication n= with n=** | | | **Headache** | **Fever** | **Loss of smell-taste** | **Sore throat** | **Shortness of breath** | **Joint-muscle pain** | **Dry cough** | **COVID-19 ^a^** |
| --- | --- | --- | --- | --- | --- | --- | --- | --- | --- | --- |
|  |  |  | **629** | **80** | **97** | **493** | **203** | **230** | **556** | **142** |
|  |  |  |  |  |  | **(%)** |  |  |  |  |
| **Statins** | **3296** | **no** | 18 | 2 | 3 | 14 | 5 | 9 | 16 | 4 |
|  | **358** | **yes** | 8 | 1 | 2 | 7 | 6 | 8 | 11 | 1 |
|  |  |  |  |  |  |  |  |  |  |  |
| **NSAID** | **3442** | **no** | 17 | 2 | 3 | 13 | 5 | 9 | 15 | 4 |
|  | **212** | **yes** | 28 | 1 | 2 | 19 | 12 | 17 | 22 | 6 |
|  |  |  |  |  |  |  |  |  |  |  |
| **Thyroid** | **3069** | **no** | 17 | 2 | 3 | 14 | 6 | 9 | 15 | 4 |
| **medication** | **585** | **yes** | 19 | 1 | 2 | 11 | 5 | 9 | 15 | 4 |
|  |  |  |  |  |  |  |  |  |  |  |
| **Omeprazole/** | **3255** | **no** | 17 | 2 | 3 | 13 | 5 | 9 | 15 | 4 |
| **Pantoprazole** | **399** | **yes** | 18 | 3 | 2 | 14 | 7 | 11 | 15 | 4 |
|  |  |  |  |  |  |  |  |  |  |  |
| **Metamizole** | **3527** | **no** | 17 | 2 | 3 | 13 | 5 | 9 | 15 | 4 |
|  | **127** | **yes** | 32 | 3 | 6 | 19 | 14 | 20 | 24 | 6 |
|  |  |  |  |  |  |  |  |  |  |  |
| **Antihypertensives (all)** | **2560** | **no** | 19 | 3 | 3 | 15 | 5 | 9 | 15 | 4 |
|  | **1094** | **yes** | 14 | 1 | 3 | 10 | 6 | 9 | 17 | 3 |
|  |  |  |  |  |  |  |  |  |  |  |
| **Furosemide** | **3462** | **no** | 17 | 2 | 3 | 14 | 6 | 9 | 15 | 4 |
| **HCT** | **192** | **yes** | 13 | 1 | 3 | 6 | 5 | 8 | 18 | 1 |
|  |  |  |  |  |  |  |  |  |  |  |
| **Cortisone** | **3485** | **no** | 17 | 2 | 3 | 13 | 5 | 9 | 15 | 4 |
|  | **169** | **yes** | 23 | 3 | 2 | 18 | 12 | 15 | 20 | 7 |
|  |  |  |  |  |  |  |  |  |  |  |
| **DMARDS** | **3598** | **no** | 17 | 2 | 3 | 13 | 5 | 9 | 15 | 4 |
|  | **56** | **yes** | 16 | 2 | 2 | 25 | 14 | 9 | 20 | 5 |
|  |  |  |  |  |  |  |  |  |  |  |
| **Antihistamines** | **3415** | **no** | 17 | 2 | 3 | 13 | 5 | 9 | 15 | 4 |
|  | **239** | **yes** | 23 | 2 | 3 | 15 | 8 | 10 | 16 | 5 |
|  |  |  |  |  |  |  |  |  |  |  |
| **Biologics** | **3512** | **no** | 17 | 2 | 3 | 14 | 6 | 9 | 15 | 4 |
|  | **142** | **yes** | 28 | 3 | 1 | 13 | 5 | 11 | 16 | 1 |
|  |  |  |  |  |  |  |  |  |  |  |
| **Hydroxy-** | **3626** | **no** | 17 | 2 | 3 | 13 | 6 | 9 | 15 | 4 |
| **chloroquine** | **28** | **yes** | 32 | 4 | 0 | 25 | 11 | 7 | 25 | 7 |
|  |  |  |  |  |  |  |  |  |  |  |
| **Subgroup ARBs** | **3615** | **no** | 17 | 2 | 3 | 14 | 6 | 9 | 15 | 4 |
|  | **39** | **yes** | 13 | 0 | 0 | 5 | 8 | 8 | 18 | 3 |
|  |  |  |  |  |  |  |  |  |  |  |
| **Subgroup ACEis** | **3242** | **no** | 18 | 2 | 3 | 14 | 5 | 9 | 15 | 4 |
| **e.g. ramipril** | **412** | **yes** | 13 | 1 | 1 | 11 | 6 | 9 | 16 | 3 |
|  |  |  |  |  |  |  |  |  |  |  |
| **Subgroup Beta-** | **3217** | **no** | 18 | 2 | 2 | 14 | 6 | 9 | 15 | 4 |
| **Blockers** | **437** | **yes** | 13 | 2 | 5 | 11 | 6 | 9 | 18 | 4 |
|  |  |  |  |  |  |  |  |  |  |  |
| **Subgroup Ca-** | **3445** | **no** | 17 | 2 | 3 | 14 | 6 | 9 | 15 | 4 |
| **channelblockers e.g. amlodipine** | **209** | **yes** | 17 | 1 | 4 | 11 | 5 | 7 | 18 | 2 |

**^a^** = COVID-19 defined by either positive PCR-test or four out of seven positive symptoms. Numbers of participants taking the specific medication in row two, numbers of participants having a symptom line two.
